# Parainfluenza 3-Induced Cough Hypersensitivity in the Guinea Pig Airways

**DOI:** 10.1371/journal.pone.0155526

**Published:** 2016-05-23

**Authors:** Eric J. Zaccone, TinaMarie Lieu, Yukiko Muroi, Carl Potenzieri, Blair E. Undem, Peisong Gao, Liang Han, Brendan J. Canning, Bradley J. Undem

**Affiliations:** 1 Department of Medicine, Division of Allergy and Clinical Immunology, Johns Hopkins University, School of Medicine, Baltimore, Maryland, United States of America; 2 The Solomon H. Snyder Department of Neuroscience, Center of Sensory Biology, the Johns Hopkins University School of Medicine, Baltimore, Maryland, United States of America; St. Jude Children's Research Hospital, UNITED STATES

## Abstract

The effect of respiratory tract viral infection on evoked cough in guinea pigs was evaluated. Guinea pigs were inoculated intranasally with either parainfluenza type 3 (PIV3) and cough was quantified in conscious animals. The guinea pigs infected with PIV3 (day 4) coughed nearly three times more than those treated with the viral growth medium in response to capsaicin, citric acid, and bradykinin. Since capsaicin, citric acid, and bradykinin evoked coughing in guinea pigs can be inhibited by drugs that antagonize the transient receptor potential cation channel, subfamily V, member 1 (TRPV1), it was reasoned that the virally-induced hypertussive state may involve alterations in TPRV1 activity. PIV3 infection caused a phenotypic switch in tracheal nodose Aδ “cough receptors” such that nearly 50% of neurons began to express, de novo, TRPV1 mRNA. There was also an increase TRPV1 expression in jugular C-fiber neurons as determined by qPCR. It has previously been reported that tracheal-specific nodose neurons express the BDNF receptor TrkB and jugular neurons express the NGF receptor TrkA. Jugular neurons also express the artemin receptor GFRα3. All these neurotrophic factors have been associated with increases in TRPV1 expression. In an ex vivo perfused guinea pig tracheal preparation, we demonstrated that within 8 h of PIV3 infusion there was no change in NGF mRNA expression, but there was nearly a 10-fold increase in BDNF mRNA in the tissue, and a small but significant elevation in the expression of artemin mRNA. In summary, PIV3 infection leads to elevations in TRPV1 expression in the two key cough evoking nerve subtypes in the guinea pig trachea, and this is associated with a hypertussive state with respect to various TRPV1 activating stimuli.

## Introduction

Respiratory virus infections are associated with increased bronchial reactivity and exacerbations of asthma [1–3] [4] [5]. In laboratory animals, viral infections lead to changes in vagal sensory and autonomic neural pathways in manners that could help to explain mechanistically viral-induced increased airway responsiveness [6–12]. The sensitivity to capsaicin-induced cough in subjects and guinea pigs has also been found to increase in the presence of a respiratory tract viral infection, and viral infections can result in a persistent post-viral nagging cough [5,13,14]. Here we have focused on potential mechanisms by which respiratory viral infections can lead to such increases in cough sensitivity in guinea pigs.

The two major sensory nerves types involved with coughing in guinea pigs are nodose ganglion derived Aδ mechanosensory nerves, and jugular ganglion derived C-fibers [15] [16]. In the trachea, both of these nerves types terminate superficially near the airway lumen [17,18]; a position likely to be influenced by products released from virally infected epithelial cells.

Neurotrophins are a family of peptides that promote survival, growth, and differentiation of neurons. It is well established that respiratory viral infections are associated with elevations of neurotrophins in the airways [19] [20]. Neurotrophic factors, such as NGF and brain derived neurotrophic factor (BDNF), interact with receptors expressed on sensory nerve endings in a peripheral target tissue, and via retrograde transport mechanisms, cause changes in gene expression in nerve cell bodies situated in the distant sensory ganglion[21]. Our previous findings reveal that TrkB and GFRα1, high affinity receptors for BDNF and glial-derived neurotrophic factor (GDNF), are highly expressed by nodose Aδ fiber neurons terminating in the extrapulmonary airways. In addition to TrkA, we have demonstrated that GFRα1 and GFRα3, receptors for the artemin (member of the GDNF ligand family) are expressed on jugular C-fibers terminating in the extrapulmonary airways[22].

In this study, we addressed the hypothesis that virus infection leads to an increase in cough sensitivity and this may be secondary to phenotypic changes in airway-specific nodose Aδ and jugular C-fiber neurons. This sensory neuromodulation may potentially be the result of PIV3-dependent increases in epithelial-derived growth factors; however this requires further investigation.

## Materials and Methods

### Animals

All experiments were performed with approval from the Johns Hopkins University Animal Use and Care Committee. Young male Hartley guinea pigs (200g to 250g) were obtained from Hilltop Laboratory Animals, Inc. (Scottsdale, PA, USA). The animals were acclimated before use and were housed in filtered ventilated cages on 7097 Teklad corn cob bedding, provided HEPA-filtered air, 2040 Teklad Global Guinea Pig Diet and tap water ad libitum, under controlled light cycle (12 h light) and temperature (22–25°C) conditions. All animals were closely monitored and any animal that displayed behaviors indicating excessive pain or infection was euthanized immediately via CO_2_ asphyxiation.

### Inoculation of guinea pigs with PIV3

The PIV3 virus was prepared in Hela human cell line. The virus and identical vehicle control were prepared and by Virasource (Research Triangle Park, NC, USA). Animals were lightly anesthetized by injection (i.p.) of ketamine (25 mg/kg) and xylazine (1.25 mg/kg) dissolved in phosphate buffer saline (PBS) and held in a supine position with the head firmly supported. The virus or virus-free medium was then pipetted into the nostrils by using a Gilson, Inc. (Middleton, WI, USA) pipette. Each guinea pig was given a 150 μl inoculation, 15 μl in one nostril, which was then repeated after 2 min in the other nostril until the full dose was given.

### Cough and airway reactivity in vivo

Four days after the virus injection, coughing experiments in conscious guinea pigs were performed as described previously (Canning et al., 2004; Muroi et al., 2011). Briefly, animals were placed in a cylindrical plethysmograph which was continuously filled with room air using an air pump while respiratory activity was monitored by sound and pressure changes within the chamber. We assessed bradykinin (BK) aerosols (0.1 mg/ml), and increasing concentrations of capsaicin (0.1, 1, 3, and 10 μM; Sigma, St. Louis, MO, USA) and citric acid (CA; 0.01, 0.1, 0.3 M), which was dissolved in saline and delivered to the chamber by an ultrasonic nebulizer (generates ∼5 μm particle size) via plastic tubing for 3 min at each dose, followed by an additional 2 min observation period. All physiological parameters were recorded digitally using a Biopac data acquisition system (Biopac, Santa Barbara, CA, USA). Continuous visual monitoring and the sound and pressure recordings were used to quantify coughing. The aerosol concentrations of each agent used were chosen based on published evidence for their effects on cough and preliminary experiments.

### Retrograde labelling and cell dissociation

Guinea pigs were anesthetized by injection (i.p) of ketamine (50 mg/kg) and xylazine (2.5 mg/kg) dissolved in phosphate buffer saline (PBS). Afferent sensory neurons of the trachea were retrogradely labeled using 5 μl fluorescent retrograde tracer Dil (Invitrogen, Carlsbad, CA, USA) solution (1% in DMSO then diluted 1:10 in PBS) as explained in [12]. Briefly, tracheotomy was performed and the dye was injected into two sites along the upper tracheal submucosa. The incisions were sutured and animal was allowed to recover for approximately 1 week.

Animals were sacrificed and both the jugular and nodose ganglia were dissected and cleaned of connective tissue. Isolated ganglia were incubated in the enzyme buffer (2 mg ml^−1^ collagenase type 1A and 2 mg ml^−1^ dispase II in Ca^2+^-, Mg^2+^-free Hanks' balanced salt solution) for 30 min at 37°C. Neurons were dissociated by trituration with three glass Pasteur pipettes of decreasing tip pore size, then washed by centrifugation (three times at 1000 g for 2 min) and suspended in L-15 medium containing 10% fetal bovine serum (FBS). The cell suspension was transferred onto poly d-lysine/laminin-coated coverslips. Neuron-attached coverslips were incubated (37°C) for 2 h before use.

### Cell picking

Coverslips of retrogradely labelled, dissociated neurons were perfused continuously by PBS and identified using fluorescence microscopy as described in Lieu et al., 2011. In brief, single cells were harvested into a glass-pipette (tip diameter 50–150 μm) pulled with a micropipette puller (Model P-87, Sutter Instruments Co., Novato, CA, USA) by applying negative pressure. The pipette tip containing the cell was broken into a PCR tube containing RNase Inhibitor (1 μl, RNAseOUT, 2 U/l, Invitrogen), immediately frozen, and stored at −80°C. Only the neurons free of debris or attached cells were collected. From one coverslip, one to four cells were collected.

### Single-cell RT-PCR

First-strand cDNA was synthesized from single neurons by using the Super-Script III CellsDirect cDNA Synthesis System (Invitrogen) according to the manufacturer's recommendations. Samples were lysed by the addition of 10 μl of resuspension buffer and incubated at 75°C for 10 min. Each sample was next treated with 5 μl of DNase I and 1.6 μl with 10× DNase I buffer. After 5 min of incubation at room temperature of DNase treatment, 1.2 μl of 25 mM EDTA was added to each sample and placed in a thermocycler for an incubation at 70°C for 5 min. Subsequently, 1 μl of oligo (dT), random primers (Invitrogen), and 10 mM dNTP mix were added to each sample and then incubated at 70°C for another 5 min. The final addition of 5× RT buffer (6 μl), RNase OUT (1 μl), and 0.1 M DTT (1 μl) were added to each sample. Water was added to the sample for the negative RT control while the remaining sample was reverse transcribed by adding Superscript III Reverse Transcriptase for cDNA synthesis. Each tube was transferred to the thermal cycler preheated to 50°C for 50 min with an inactivated step set at 85°C for 5 min. The reaction was chilled at 4°C, and cDNA was stored at −20°C until PCR amplification.

The PCR reaction mixture contained 0.5 U of HotStar Taq Polymerase, 2.5 mM MgCl2, and 10× PCR buffer (Qiagen, Valencia, CA, USA). Additionally 10 mM dNTP and selected primers (Invitrogen) were added to the reaction mixture in a volume of 2 μl. The PCR reaction conditions were on a 50-cycle basis with initial activation at 95°C for 15 min, denaturation at 94°C for 30 s, annealing at 60°C for 30 s, and extension at 72°C for 1 min followed by a final extension at 72°C for 10 min. Products were visualized in ethidium bromide-stained 1.5% agarose gels with a 50- or 100-bp DNA ladder.

### Trachea homogenization and RT-PCR

Confirming the presence of PIV3 in the infected airway (day 4), guinea pig tracheas were removed and snap-frozen in liquid nitrogen for RNA extraction. The trachea was homogenized and spun through a QIAshredder (Qiagen), followed by RNA extraction using the RNeasy Mini Kit (Qiagen) according to the manufacturer's instructions. RNA was reverse transcribed as previously described. Primers used to confirm the presence of the virus are listed in Table 1.

**Table 1:** Sequence of primers used for PCR analysis of PIV3.

Gene	Primer	Sequence (5′-to 3′)
β-actin	Forward	TGGCTACAGTTTCACCACCA
	Reverse	GGAAGGAGGGCTGGAAGA
Nucleocapsid Protein 1	Forward	CGGTGGAGCTATCATTCCTG
	Reverse	CCCTTTGTGCATGTTGTTTCT
Nucleocapsid Protein 2	Forward	GCAGGTCTCGCTTCATTCTT
	Reverse	ACTCCCATTGCATAGCTCCA
Fusion Protein	Forward	GGGAGTAGCAACCTCAGCAC
	Reverse	TTGATGGCACGATTTCTTTG
Hemagglutinin-Neuraminidase 1	Forward	TGCTGGTAATGAGCTGGAGA
	Reverse	TGCCATTTGGATCTTTTCTGT
Hemagglutinin-Neuraminidase 2	Forward	CCGGGAAAACACAGAGAGAC
	Reverse	TTCCTTCTGACCCCCAGTAA

### Quantitative PCR (qPCR)

Total cellular RNA was extracted from the ganglia or trachea using the RNeasy Micro Kits (Qiagen) according to the manufacturer's protocol. RNA was reverse-transcribed into cDNA using SuperScript^®^ III First-Strand Synthesis System (Invitrogen). Quantitative real-time PCR was performed on cDNA using a StepOnePlus Real-Time PCR System (Thermo Fisher Scientific, Waltham, MA, USA) with the SYBR Green detection method. The raw threshold cycle (CT) values were analyzed by the 2−ΔΔCt method to determine normalized expression ratios of target genes. The primers used are listed in Tables 2 and 3.

**Table 2:** Sequence of primers used for analysis of guinea pig ganglia receptor transcripts.

Gene	Primer	Sequence (5′-to 3′)
GAPDH	Forward	CCAGAACATCATCCCCGCAT
	Reverse	TGTCATCGTATTTGGCCGGT
TRPV1	Forward	CCAACAAGAAGGGGTTCACA
	Reverse	ACAGGTCATAGAGCGAGGAG

**Table 3:** Sequence of primers used for qPCR analysis of guinea pig neurotrophin transcripts.

Gene	Primer	Sequence (5′-to 3′)
GAPDH	Forward	CCAGAACATCATCCCCGCAT
	Reverse	TGTCATCGTATTTGGCCGGT
BDNF	Forward	TAAAAAGACGGCCGTGGACA
	Reverse	TTGTCTATGCCTCTGCAGCC
Artemin	Forward	GGGAGCTCCTGGTGTTGATAG
	Reverse	ACAGAAGACTCTGTGACGCT
NGF	Forward	GCCAAGGGAGCAGCTTTCTA
	Reverse	TTCGAAGGGCTGTGTCAAGG

### Immunofluorescence staining

Four days following PIV3 infection, guinea pigs were killed by an overdose of pentobarbital (150 mg/kg, i.p.), transcardially perfused with 4% formaldehyde in PBS (pH 7.4; 60 ml) and the trachea was removed, cleaned of connective tissue and fixed overnight in (4°C). Cryostat sections (10 microns) of tracheas were blocked with a solution containing 10% donkey serum, 1% BSA, and 0.1% Tween 20 in PBS, then incubated (4°C) overnight in primary antibodies diluted in PBS containing 1% BSA and 0.5% Triton X-100, rinsed, and then covered in species-specific secondary antibodies conjugated with Alexa dyes 488 (Invitrogen-Molecular Probes) recognizing species-specific primary antibodies. We used rabbit anti-PIV3 (MAB855-1; EMD Millipore, Billerica, MA, USA). For negative controls, separate sections were processed similarly, but with secondary antibody only. Serial sections of the trachea from control and PIV3-treated animals were analyzed. The slides were evaluated using micrographs taken by fluorescent microscope (Olympus BX-50) equipped with a camera (Q-Imaging Retiga Exi camera); a micrograph field of view of the entire stained section was taken.

### Isolated perfused trachea

Guinea pigs were euthanized by CO_2_ asphyxiation. A 4-cm segment of trachea was removed and cleaned of connective tissue. The airway was mounted and tied between two pieces of tubing at its *in situ* length and placed in an extraluminal (EL) bath containing Krebs bicarbonate buffer (NaCl, 118 mM; KCl, 5.4 mM; NaH2PO4, 1.0 mM; MgSO4, 1.2 mM; CaCl2, 1.9 mM; NaHCO3, 25.0 mM; dextrose, 11.1 mM) containing 1% BSA. In one side of the isolated trachea, the tubing acted as a catheter in the lumen, which was connected to peristaltic pump (Instech Laboratories, Plymouth Meeting, PA, USA), which perfused at a constant rate (200 μl/min is ideal) with 1% BSA in Krebs from a separate bath, the intraluminal (IL) bath. The tubing tied in the other end of the trachea recycled the Krebs buffer into the IL bath. The pump regulated the flow of Krebs buffer flowing through the lumen of the trachea. Krebs in the EL baths was replaced at 30-min intervals with fresh solution. Krebs temperature was 37°C and was aerated with 95% O_2_/5% CO_2_ to give pH 7.4. Different dilutions (1/10, 1/100, 1/1000) from viral stock solution PIV3 (2x10^7^ pfu/ml) of virus for different time periods (8, 12, and 24 hours) were administered to the IL bath and mRNA expression evaluated. Our preliminary studies indicated perfusions for 8 h at a dilution factor of 1/100, resulted in optimal viral infection to increase neurotrophic factors. These studies also demonstrated that the guinea pig tracheal epithelium remains intact, and the tissue viable for at least 24 hours.

### Drug preparations and applications

Drugs and solutions were purchased from Sigma Chemicals Co. (St Louis, MO, USA) and dissolved in 0.9% saline. Capsaicin (Sigma) was made up as a 10 mM stock solution with ethanol and diluted in saline solution to a final concentration 1 mM. BK and CA (Sigma) was made up as a 1 mM stock solution with saline.

### Statistical Analysis

The results are expressed as mean ± SEM. Significant differences were evaluated using a t-test for qPCR analysis in addition to in vivo studies using TRPV1 agonists. *n* is the number of animals in each experiment. *p*<0.05 was considered significant.

## Results

### PIV3 infection

**Fig 1:**
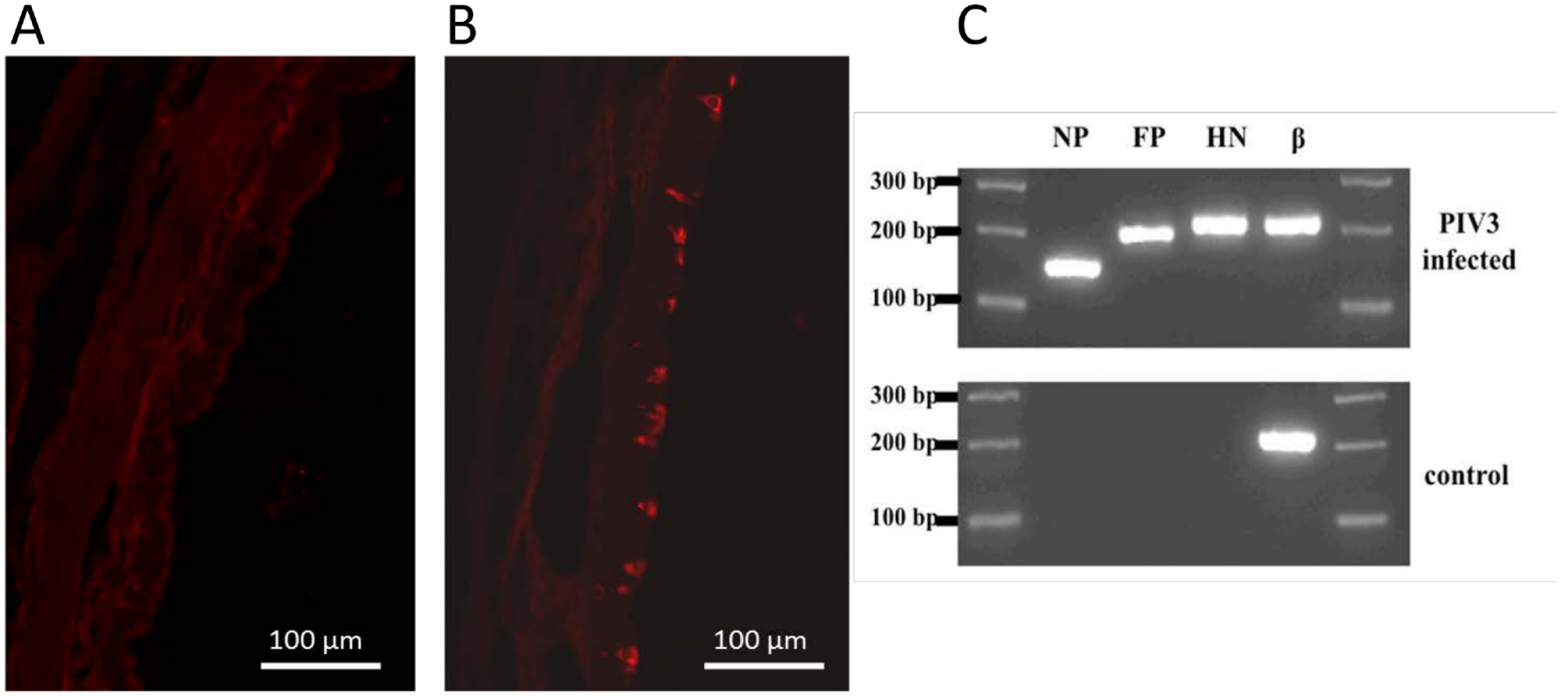
PIV3 infection of epithelium in guinea pig trachea 4 d after inoculation with virus or vehicle (control). A) PIV3 staining of tracheal epithelium from a vehicle-treated control animal. B) PIV3 staining of tracheal epithelium following viral infection in vivo. Bar = 100 μm. C) Each lane represents RT-PCR amplification with primers for PIV3 genes: nucleocapsid protein (NP), fusion protein (FP), hemagglutinin-neuraminidase (HN), and a positive control- guinea pig β-actin (β).

It has been demonstrated that guinea pigs airways are readily infected by the human PIV3 [23]. To ensure successful PIV3 infection of the guinea pig airways, we employed immunohistochemistry and PCR to PIV3 staining and gene expression in the epithelium. Compared to a control, PIV3 immunoreactivity is demonstrated in the airway epithelium 4 days following virus infection in vivo (Fig 1A & 1B). Expression of PIV3 RNA in guinea pig trachea after inoculation with virus was compared to controls. Each lane represents PCR amplification with primers for PIV3 genes: nucleocapsid protein (NP), fusion protein (FP), hemagglutinin-neuraminidase (HN), and positive control β-actin (β) (Fig 1C), confirming the presence of the virus 4 d after inoculation. These genes were not express in untreated animals. We evaluated tracheal neutrophils in two virus treated and three vehicle treated animals on day 4 after inoculation. There was a mild increase in tissue neutrophils that averaged 4.5 ± 0.4 neutrophils/tracheal section compared to 0.9 ± 0.3 neutrophils/section virus vs vehicle inoculated animals, respectively (P <0.01). we did not evaluate the inflammation in the lungs of the animals, but based on other studies it is likely that intrapulmonary airways are also likely infected (14).

### PIV3 infection and cough sensitivity

**Fig 2:**
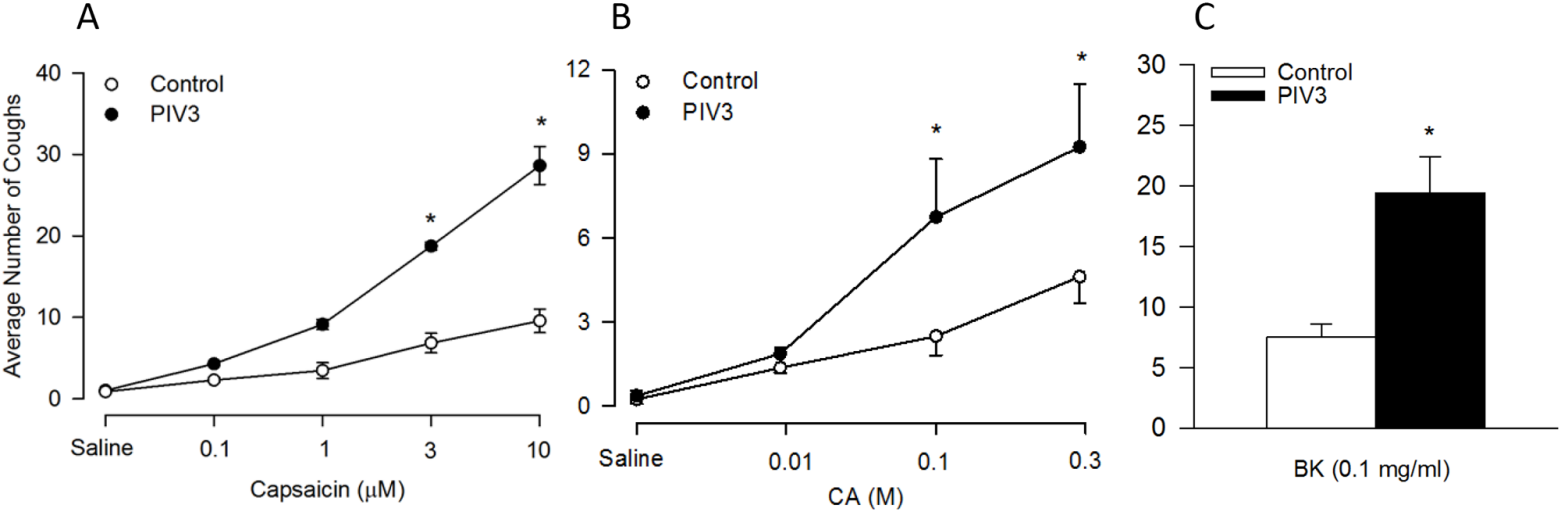
Guinea pig cough responses to inhaled capsaicin (0.1, 1, 3, 10 μM), citiric acid (CA; 0.01, 0.1, 0.3 M) and bradykinin (BK; 0.1 mg/ml) 4 d after viral inoculation. Cough was confirmed in conscious guinea pigs based on changes in pressure and visual confirmation. Comparison of the average number of coughs between infected animals and controls were compared. A) Capsaicin: Control, n = 4; PIV3: n = 4. B) CA: Control, n = 8; PIV3, n = 8. D) Bradykinin: Control, n = 10; PIV3, n = 10. *Significantly different from control (p<0.05).

There was little or no background coughing in control or virally treated animals. Capsaicin inhalation (0.1–10 μM) evoked coughing above background, and at the larger doses (>3μM) this was markedly increased in PIV3 infected animals (Fig 2A). Citric acid (0.01–0.3 M) inhalation consistently evoked coughing, and as with capsaicin this was markedly enhanced in the virus treated guinea pigs (Fig 2B). PIV3 infection also markedly potentiated coughing evoked by bradykinin (Fig 2C). In previous studies we have noted that bradykinin-induced cough desensitized upon repeated challenge so we elected only to quantify the cough to one dose (0.1 mg/ml) of bradykinin. Overall, coughs evoked by bradykinin more than doubled during PIV3 infection (19±4 vs. 39±9 coughs; n = 8/ treatment PIV3 infected animals). The influence of PIV3 infection was even more noticeable when the time course of bradykinin-induced coughing was evaluated. The onset of bradykinin evoked coughing was faster in the infected animals, with 0.6±03 and 9±3 coughs occurring over the first 2 minutes of challenge in control and infected guinea pigs, respectively (n = 8/ treatment group; p<0.02). Although a comparable amount of coughing during the ensuing 2 minutes of challenge was observed (15±3 and 13±5 coughs, respectively), a period of the challenges when paroxysms of cough were frequent. Interestingly uninfected animals typically adapted to the stimulus and stopped coughing thereafter, despite 6 additional minutes of continuous bradykinin challenge (3±1 coughs), while coughing persisted in the virally infected animals (17±2 coughs; p<0.001).

### TRPV1 mRNA expression

**Fig 3:**
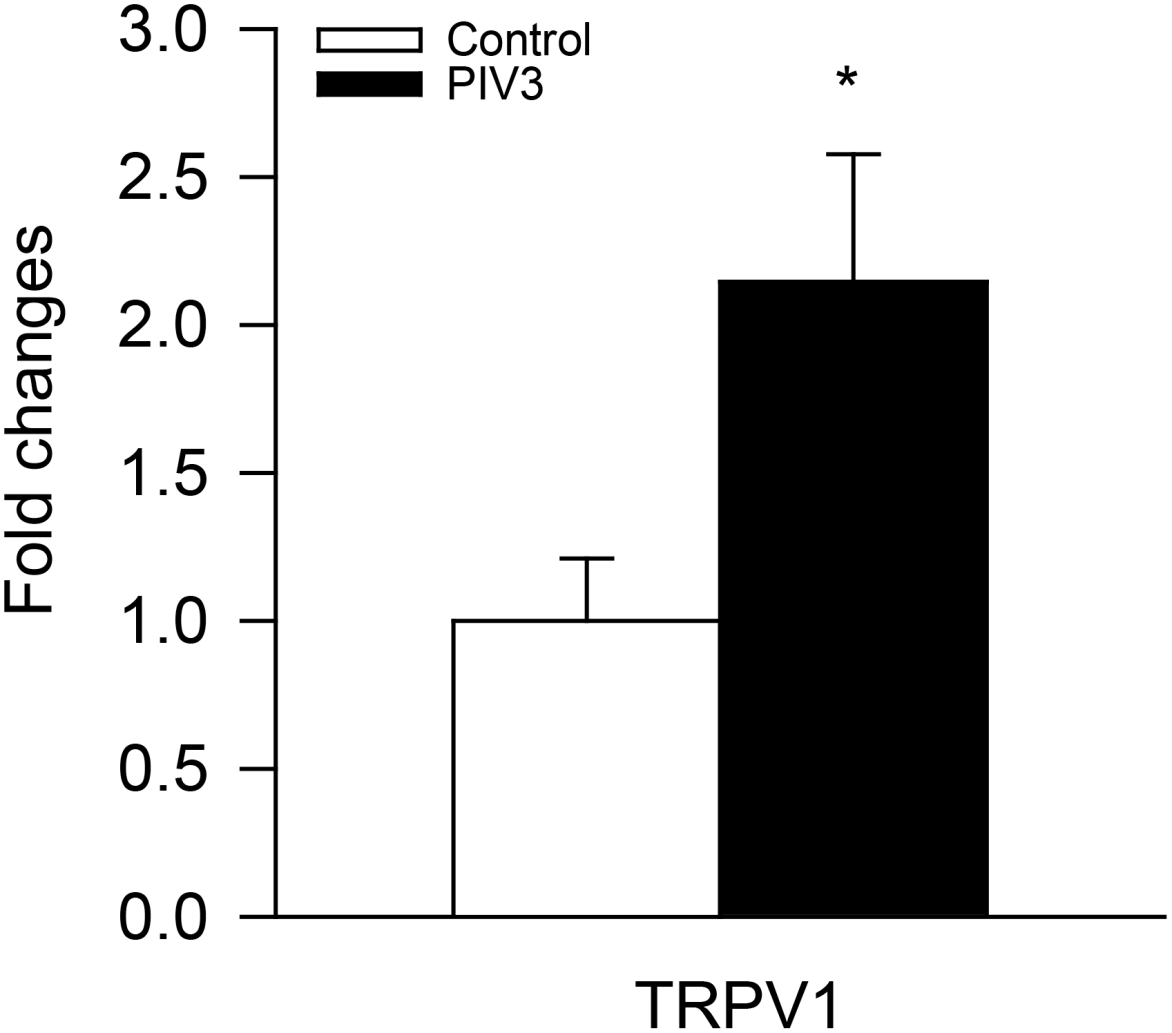
TRPV1 expression in the jugular ganglia in control animals compared to those inoculated with PIV3 (day 4). Increased expression of TRPV1 (qPCR) following PIV3 infection for 4 d. Control: n = 7, 14 ganglia; PIV3: n = 7, 14 ganglia. *Significantly different from control (p<0.05).

**Fig 4:**
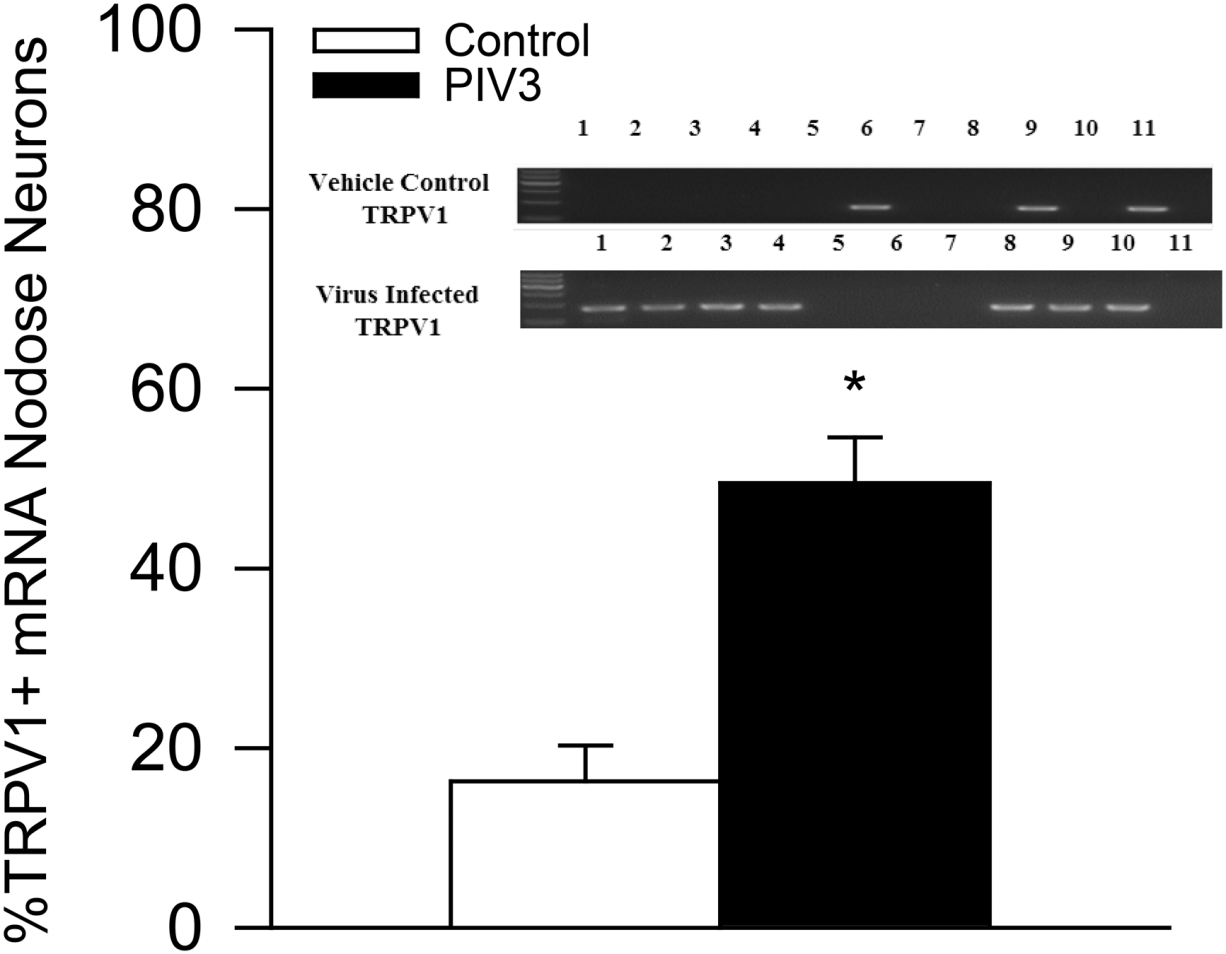
Percentage of guinea pig tracheal-specific nodose neurons that express TRPV1 in controls compared to those inoculated with PIV3 (day 4). Top, representative mRNA expression of TRPV1 from neurons. Bottom, total TRPV1 mRNA expression in infected animals (49.48 ±5.1%) compared to controls (16.28 ±4.0%). Control: n = 11, 86 neurons; PIV3: n = 10, 97 neurons. *Significantly different from control (p<0.05).

Since capsaicin, bradykinin, and citric acid-evoked coughing in guinea pigs have all been found to depend totally or in part on TRPV1 [24–26], we evaluated the TRPV1 expression in vagal sensory ganglia in vehicle vs PIV3 infected (day 4) animals. Based on qPCR assessment there was over a 2-fold increase in TRPV1 mRNA expression in jugular ganglia (P<0.05; Fig 3), but no significant difference in nodose ganglia. Unlike the jugular ganglia, only a small fraction of nodose neurons innervate the large airways, we reasoned that any signal from the infected airway nerves might be diluted by gene expression in the non-airway specific C-fibers. To evaluate TRPV1 expression specifically in airway-specific nodose neurons we evaluated, using single-cell PCR analysis, gene expression in individual nodose neurons retrogradely labeled from the trachea. We have previously reported that whereas the jugular fibers innervating the trachea are nearly all TRPV1 expressing and capsaicin sensitive, the nodose neurons project mainly capsaicin-insensitive Aδ fiber nerves [27]. Accordingly only a small percentage of tracheal specific nodose neurons express TRPV1 mRNA[28]. Consistent with this, in control animals only about 16% of the tracheal-specific nodose neurons expressed TRPV1 mRNA (Fig 4). There was phenotypic switch in nodose neurons innervating trachea of guinea pigs inoculated 4 days earlier with PIV3 such that about 50% began expressing TRPV1 (P <0.01; Fig 4).

### PIV3 and neurotrophic factors

**Fig 5:**
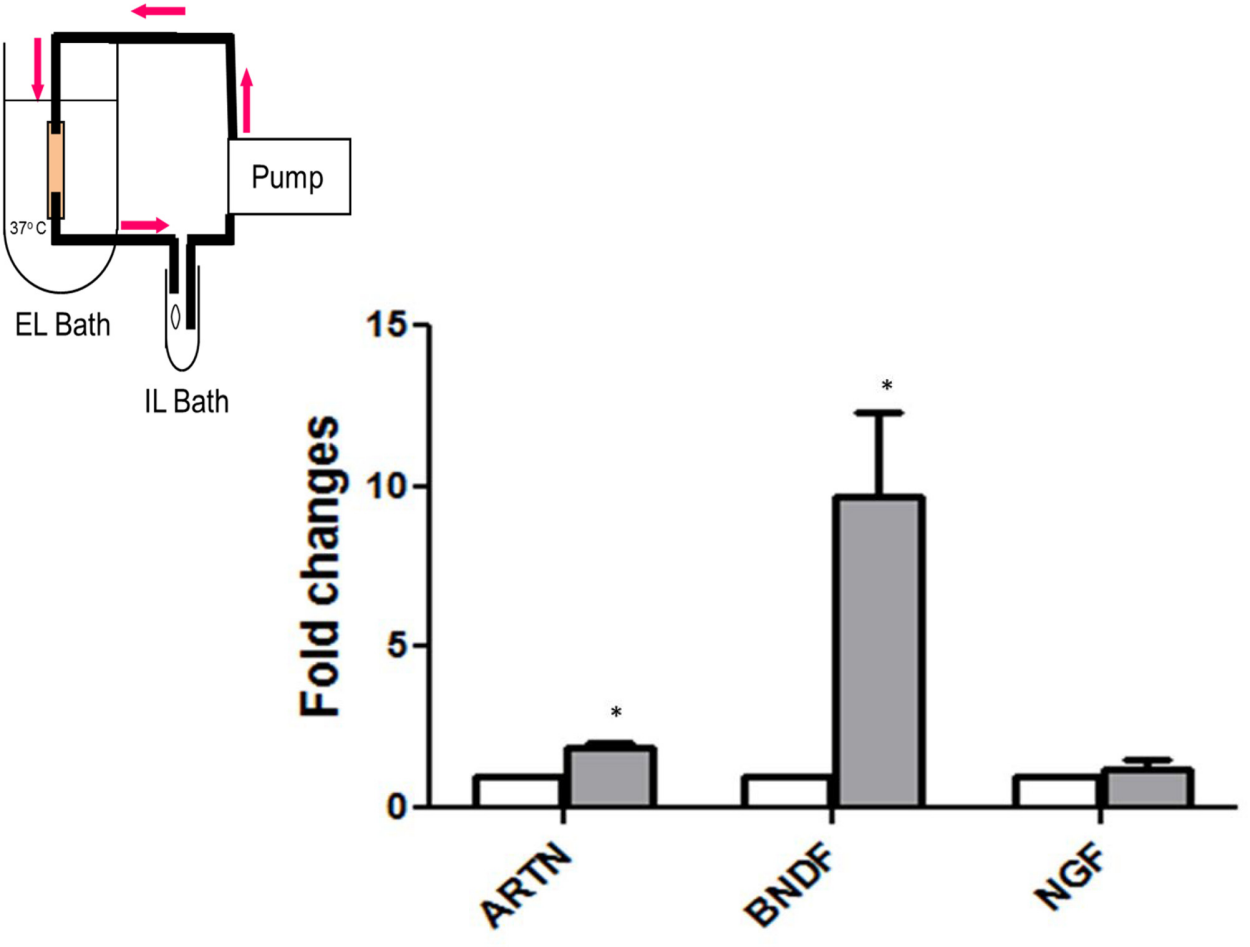
Vehicle or viral infections of the trachea occurred ex vivo for qPCR analyses of neurotrophin levels. Upper left, a schematic of the isolated, perfused guinea pig airway apparatus used to virally infect intact epithelium. Lower right graph demonstrates an increased expression of BNDF and artemin (ARTN) in trachea following infection with 1/100 diluted PIV3 virus (stock = 2x10^7^ pfu/ml) for 8 h. The data are the average of 4 separate experiments. *Significantly different from control (p<0.05).

A change in gene expression at the distant cell bodies induced by virally infected airway epithelium most likely involves the interaction of neurotrophic factors with the nerve terminals in the trachea [21]. In order to investigate the direct role of virus in upregulating neurotrophin expression in the airway epithelium independently of the cellular infiltration associated with the inflammatory response, we evaluated the effect of virus on the tracheal tissue *ex vivo*. Previous studies have found that PIV3 efficaciously and rapidly infects tracheal/bronchial epithelial cells *ex vivo* with replication near maximal within the first 24 hours of exposure [29]. Different PIV3 dilutions (1/10, 1/100, 1/1000) from viral stock (2x10^7^ pfu/ml) were used in preliminary studies to determine the optimal viral concentration and time point (8 h, 12 h, 24 h) for the upregulation of various neurotrophic factors (data not shown). The optimal concentrations proved to be the 1:100 dilution (2x10^5^ pfu/ml). Perfusing the trachea with PIV3 caused a ~10-fold increase in BDNF mRNA expression within 8 h (P<0.05, n = 4 experiments). There was also a significant increase in artimen mRNA in the tissue (Fig 5). There was no change in NGF at this time period (Fig 5). We could not detect glial cell line-derived neurotrophic factor (GDNF) in control or virally treated trachea; data not shown).

## Discussion

Parainfluenza viruses are non-segmented negative-strand RNA viruses that cause bronchiolitis, coughing, and augment pre-existing reactive airway diseases such as asthma [29–33]. It has been demonstrated by us and others that PIV infection typically peaks in days 3–5 of infection and that there is a maximal increase in cough at this time point[9] [14]. We found that PIV3 infection (day 4) of guinea pig airways leads to a strong enhancement of cough evoked by capsaicin, bradykinin, and citric acid. The observation that PIV3 infection increases cough in response to capsaicin is consistent with the recent findings of Ye et al. [14].

Chemically evoked coughing in conscious healthy guinea pigs is secondary to stimulation of vagal C-fibers [15] [16]. These C-fibers by in large arise from the jugular ganglia. Capsaicin strongly stimulates jugular C-fibers in the trachea, but not nodose Aδ cough receptors [15]. The receptor for capsaicin is TRPV1, and TRPV1 antagonists block capsaicin-induced activation of jugular C-fibers in the trachea and capsaicin-induced cough in guinea pigs [26,34]. TRPV1 can also be activated by acid, and blocking TRPV1 inhibits citric acid induced action potential discharge of jugular C-fiber in the trachea, and citric acid-induced cough in conscious guinea pigs [25,26,35]. Bradykinin stimulates jugular C-fibers by activation of B2 receptors [36] [37]. The bradykinin B2 receptor is a G-protein coupled receptor, and part of the stimulation mechanism downstream from stimulation of B2 receptors in jugular C-fibers is due to activation of TRPV1 [38,39]. As with citric acid and capsaicin, blocking TRPV1 inhibits bradykinin-induced coughing [40]. Based on this we reasoned that the viral infection may have led to increased capsaicin, acid and bradykinin evoked coughing by enhancing TRPV1 signaling.

It is interesting to note that in the study by Ye et al, the PIV-induced increase in capsaicin cough persisted for at least 42 days, a time when the virus was largely cleared from the airways [14]. This raises the hypothesis that viral infection may lead to sensory neuroplasticity by changing TRPV1 gene expression. Consistent with this hypothesis we noted that by day 4 of PIV3 infection there was an increase in TRPV1 expression in jugular ganglion neurons. The jugular ganglia project a large number of fibers to the large airways that are nearly uniformly capsaicin sensitive[41]. This would suggest that the response of nerve fibers already sensitive to TRPV1 stimuli, would be enhanced following the viral infection.

The nodose ganglia project mainly Aδ cough fiber receptors to the trachea [27]. Activation of these fibers can evoke coughing even in anesthetized animals[15]. In healthy animals these nerves do not respond to capsaicin and do not express TRPV1 mRNA. Only a minority of the approximately 30,000 nodose neurons innervated the large airways, as virtually all other visceral organs are also being richly innervated by nodose nerve fibers (mainly C-fibers). Since the viral infection is largely limited to larger airways, we were not surprised that we did not note an increase in TRPV1 mRNA in the nodose ganglia *per se*. However, when we focused only on tracheal specific neurons using single cell PCR, we noted that a sizeable (~50%) percentage of the neurons began to express to TRPV1 mRNA *de novo* in virally infected animals. In functional studies we have noted than only about 10% of the nodose fibers innervating the trachea are capsaicin C-fibers with ~90% being capsaicin-insensitive Aδ fibers. This means that viral infection led to a phenotypic switch in many nodose Aδ fiber neurons such that they begin to express TRPV1. These data are consistent with the idea that that activation profile of nodose Aδ cough receptors is enhanced by the viral infection to include TRPV1 activating stimuli such as capsaicin and acid. Although we did not evaluate the function of TRPV1 receptors in nodose and jugular neurons in this study, we have previously reported that allergically sensitized and challenged guinea pigs leads to a similar switch in TRPV1 mRNA expression in nodose neurons [28] and this was translated to an increase in functional TRPV1 channels within the neurons as revealed by increases in both capsaicin and acid responsiveness [42].

We have previously noted that in virally infected guinea pigs, tracheal nodose Aδ fiber neurons also begin to express neuropeptides like substance P. In non-inflamed animals these peptides are primarily expressed in jugular C-fiber neurons, and virtually never expressed in large nodose A-fiber neurons[12]. Thus, viral infection of the airways may not only increase the stimulus profile of cough fibers by increasing expression of ion channels like TRPV1, but may also qualitatively change the role mediated by sensory neuropeptides in cough reflexes. In this regard it is useful to point out that neurokinins released from central terminals of airway afferent nerves can serve to augment the cough reflex in guinea pigs [43]. A similar phenotypic switch with respect to TRPV1 induction and neuropeptide induction in nodose trachea Aδ neurons has been demonstrated with allergic inflammation[44], and this type of inflammation, as reported here with PIV3 infection, also increases capsaicin-induced and acid-induced cough in guinea pigs [45,46]. Allergic inflammation also cause TRPV1 expression *de novo* in large Aβ fiber neurons in rat nodose ganglia, and the Aβ stretch receptors (rapidly and slowly adapting receptors) acquire a sensitivity to capsaicin[47]. Whether viral infection can alter TRPV1 expression in intrapulmonary nodose neurons was not investigated here.

The mechanisms by which virally infected airway epithelial cells leads to changes in gene expression at the cell bodies located in the distant vagal ganglia likely involves the production of neurotrophic factors. Neurotrophic factors can stimulate high affinity receptors at nerve terminals that then lead to retroactive signaling that can culminate in changes in gene expression at the cell body situated in the sensory ganglion [21].

We have previously demonstrated that tracheal-specific nodose Aδ neurons express the BDNF receptor, TrkB[22]. Moreover, applying BDNF to the trachea leads to the same phenotypic switch in nodose Aδ neurons that is reported here, i.e. the de novo induction of TRPV1[28]. Moreover, the BDNF mediated induction of TRPV1 was persistent lasting for weeks. We were therefore particularly interested in whether viral infection leads to BDNF production in the airways. That infecting the isolated trachea led to a substantial induction in BDNF mRNA is consistent with, but does not prove, that TrkB activation may contribute to virally mediated TRPV1 induction in nodose neurons.

It is unlikely that BDNF contributed to the TRPV1 induction in the jugular ganglia, as jugular neurons rarely express TrkB[22]. The jugular (neural crest) derived C-fiber neurons express TrkA receptors[22], but we did not notice an increase in NGF mRNA in our studies. We have noted that members of the GFRα-RET ligands such as GDNF can also increase TRPV1 expression in vagal sensory neurons [28]. We previously reported in guinea pigs and mice that jugular C-fiber neurons express GFRα3, the receptor for artemin[22,48]. That artemin mRNA was modestly elevated in the infected trachea, supports the idea that GFRα receptors may be involved in the sensory plasticity noted, especially in the jugular ganglia. In this context it is worth noting that artemin has repeatedly been linked to increases TRPV1 expression and hypersensitivity in the somatosensory system [49–52].

Considered together, the data supports the hypothesis that viral infection of tracheal/bronchial epithelium leads to production of specific neurotrophins that can alter the nodose Aδ fiber and jugular C-fiber phenotype in a manner that would make them more sensitive to TRPV1 stimulation. Inasmuch as these are the two fiber types that cause cough in guinea pigs, the TRPV1 induction may contribute the virally mediated hypertussive response to direct TRPV1 dependent stimuli such as capsaicin, acid, and indirect TRPV1 stimuli such as bradykinin. It is recognized, however, that this conclusion remains circumstantial until specific cause-effect relationships are experimentally verified. It is unlikely that one specific mechanism will explain *en toto* the viral induced hypertussive state. There are potentially multiple neurotrophic factors involved and multiple nerve subtypes. In addition, the mechanisms may change over the course of the infection. The secondary inflammatory response to the viral infection likely leads to electrophysiological increases in the nerve terminal excitability[53], especially during the acute phase of the infection, whereas the phenotypic changes noted here may contribute more to the lingering aspect of the viral hypertussive state.

## Supporting Information

S1 File The data values behind Figs 2–5. (XLSX)

## Abbreviations

BDNF: brain-derived neurotrophic factor
BK: bradykinin
CA: Citric Acid
PIV3: parainfluenza virus type 3
TRPV1: transient receptor potential cation channel, subfamily V, member 1
